# Retraction Notice

**DOI:** 10.4103/0019-5049.76616

**Published:** 2011

The following article is being retracted as it is found to be plagiarized.

Trivedi V. Transversus abdominis plane block for an emergency laparotomy in a high-risk, elderly patient. Indian J Anaesth 2010;54:478-9.

**Editor, Indian J Anaesth**

