# ERRATUM

**Published:** 2011

**The retraction notice printed in the**

**Indian Journal of Anaesthesia Jan-Feb 2011; Vol 55; Issue 1: p 76.**

**The following article is being retracted as it is found to be plagiarized.**

**Trivedi V. Transversus abdominis plane block for an emergency laparotomy in a high-risk, elderly patient. Indian J Anaesth 2010;54:478-9.**

Should be corrected as

**The following article is being retracted as it is found to be plagiarized.**

**Trivedi V. Continuous cervical epidural analgesia for Isshiki type- I Thyroplasty. Indian J Anaesthesia 2010;54:52-5.**

The error is regretted

**Editor, Indian J Anaesth**

